# A Rare Case of Inflammatory Myofibroblastic Tumor Causing Left Pulmonary Artery Stenosis and Successfully Treated with Localized Radiotherapy in a Patient with Perinuclear Antineutrophil Cytoplasmic Antibody Vasculitis

**DOI:** 10.7759/cureus.6709

**Published:** 2020-01-20

**Authors:** Claire P Browne, Cady Zeman-Pocrnich, A. Rashid Dar, Blair Wyllie, Mariamma Joseph

**Affiliations:** 1 Internal Medicine, Schulich School of Medicine & Dentistry, Western University, London, CAN; 2 Pathology and Laboratory Medicine, London Health Sciences Centre, London, CAN; 3 Radiation Oncology, Schulich School of Medicine & Dentistry, Western University, London, CAN; 4 Internal Medicine, London Health Sciences Centre, London, CAN

**Keywords:** p-anca vasculitis, pulmonary artery stenosis, inflammatory myofibroblastic tumor, unresectable tumors

## Abstract

Inflammatory myofibroblastic tumor (IMT) of the lung is a rare neoplasm that commonly behaves in an indolent fashion and is generally treated with complete surgical excision. The management of unresectable IMT presents a significant challenge, especially in cases with multiple comorbidities, and a consensus has yet to be reached on the most appropriate first-line modality. We present a case of unresectable IMT causing severe stenosis of the left pulmonary artery in a patient on immunosuppressive therapy for perinuclear antineutrophil cytoplasmic antibody vasculitis. The patient was successfully treated with localized radiotherapy to a total dose of 45 Gy in five weeks, and has been followed for more than seven years since treatment. In this case report, we review the pertinent literature and illustrate the difficulties in diagnosing and treating rare neoplasms in a patient with significant medical comorbidities.

## Introduction

Inflammatory myofibroblastic tumor (IMT), alternately referred to as inflammatory pseudotumor (IPT) or plasma cell granuloma, is a low-grade neoplasm most commonly found in the lung, pelvis, abdomen, head and neck, and spine [1,2]. It is characterized pathologically by a mixed inflammatory infiltrate with background myofibroblastic spindle cell proliferation [1,3]. Etiology is still unclear; some theories postulate an inflammatory response to viral infection or other stimuli [1,3]. Immunohistochemistry studies indicate that approximately 50% of IMTs are positive for anaplastic lymphoma kinase (ALK), a receptor tyrosine kinase protein associated with malignancy when expressed extraneurally [1,3]. IMT occurs most frequently in the first two decades of life but may present in individuals of any age and sex; estimates of the median age of diagnosis range from 9 to 11 years [1,2].

Presentations of pulmonary IMT include dyspnea, chest pain, and constitutional symptoms [1]. Differential diagnoses include nonspecific inflammation, cryptogenic pneumonia, lymphoma, fibromatosis, fibrosclerosing lesions, inflammatory leiomyosarcoma, and IgG4-related disease [3]. Tumors are typically treated via surgical resection, and postoperative prognosis is good with a recurrence rate of less than 2% [3]. The treatment of unresectable IMTs is more challenging, and a consensus has yet to be reached on appropriate management [4]. Here, we present a rare case of unresectable IMT causing severe pulmonary artery stenosis in a patient with known perinuclear antineutrophil cytoplasmic antibody (p-ANCA) vasculitis, treated with radiotherapy. The difficulties encountered in making a definitive diagnosis illustrate the broad considerations required to discern etiology of disease in a complex patient.

## Case presentation

A 52-year-old male former smoker was admitted to hospital in March 2012 with a three-month history of increasing left-sided pleuritic chest pain and a one-month history of increasing shortness of breath on exertion. His ability to walk distances greater than half a block was limited. He had experienced orthopnea and palpitations, but denied cough, hemoptysis, or constitutional symptoms.

The patient had known p-ANCA vasculitis diagnosed in 2009 causing pauci-immune proliferative glomerulonephritis, pathologically confirmed by renal biopsy; the patient initially presented with recurrent fevers, polyarthralgia, and bullous eruption. The most recent exacerbation was in November 2011. At the time of admission, the vasculitis was controlled with prednisone and cyclophosphamide. Previous medications include azathioprine, which caused acute hepatitis and was subsequently discontinued, and methotrexate. Additionally, the patient was a 45 pack-year former smoker diagnosed with chronic obstructive pulmonary disease, for which he took tiotropium bromide and salbutamol. Pulmonary function tests from November 2011 showed a forced expiratory volume in 1 second (FEV1) of 42%, a forced vital capacity (FVC) of 78%, and a FEV1/FVC ratio of 56%, supporting an obstructive picture.

**Figure 1:**
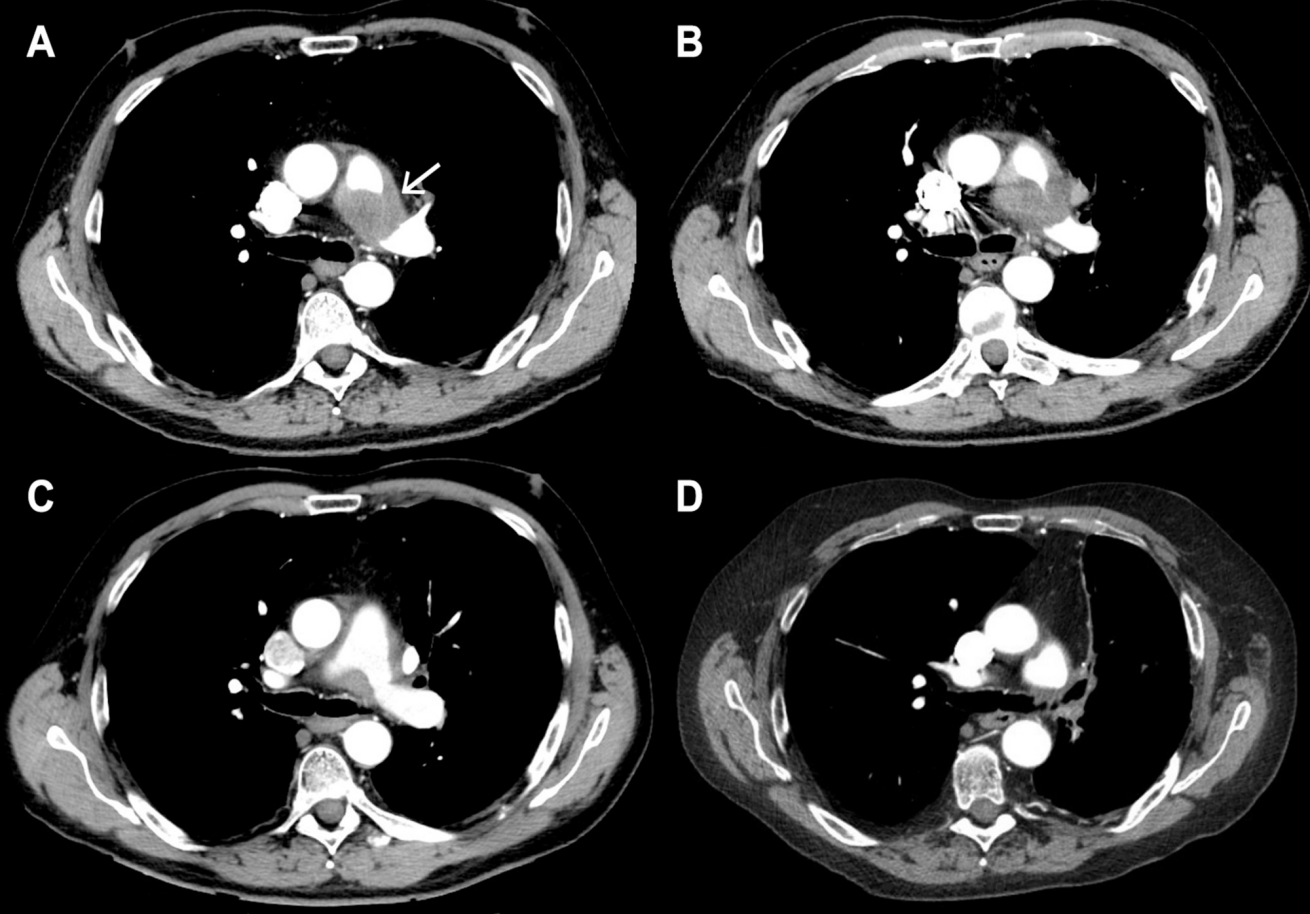
CT pulmonary angiogram images of left hilar mass causing pulmonary artery stenosis. (A) CT pulmonary angiogram from March 2012 revealed a new left hilar mass causing stenosis of the left pulmonary artery, measuring 4.5 x 3.5 cm. (B) By June 2012, the tumor had further enlarged to 5.1 x 3.9 cm. (C) One-month post-radiotherapy, the lesion had significantly reduced in size, and now measured 3.1 x 2.9 cm. (D) 7.3 years post-radiotherapy, the residual fibrotic mass remains minimal.

On initial admission, CT pulmonary angiogram (CTPA) revealed significant circumferential stenosis of the left pulmonary artery (Figure 1A). A hilar mass resulted in 80% occlusion of the artery lumen, as well as esophageal compression. This lesion was new compared to CT imaging from two years prior. A neoplastic cause could not be excluded. A segmental pulmonary embolism was also visualized and later confirmed by the V/Q scan. The presence of a mass and stenosis were correlated via MRI. Endobronchial ultrasound-guided biopsy of the mass was attempted, but due to concerns regarding proximity of the artery a sufficient sample could not be obtained. An open biopsy was performed via left thoracotomy in May 2012. This was planned as a video-assisted thoracoscopic procedure, yet was converted to an open procedure intraoperatively due to difficulty in differentiating the aorta, pulmonary artery, and tumor. Multiple samples were successfully recovered from the area enclosed by the pulmonary artery, aorta, and ligamentum arteriosum. The patient recovered and was discharged with home oxygen therapy. Despite compliance, he experienced increasing shortness of breath over the following month. Blood work revealed a normal beta human chorionic gonadotropin and lactate, and marginally elevated alpha-fetoprotein. Positron emission tomography (PET) scanning was not done at this time.

**Figure 2:**
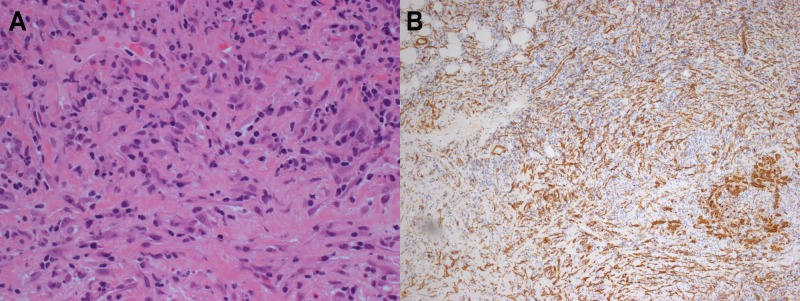
Samples of excisional biopsies showing inflammatory myofibroblastic tumor. (A) Sections were notable for proliferation of spindle cells without significant atypia and a mixed inflammatory infiltrate (hematoxylin & eosin, 400x magnification). (B) Immunohistochemical staining for smooth muscle actin reveals diffuse positivity (200x magnification).

Pathology sections of the left thoracotomy-acquired biopsies of the subaortic mass showed a proliferation of bland spindle cells with an admixed moderately dense infiltrate of lymphocytes, plasma cells, and histiocytes (Figure 2A). The lesional spindle cells had vesicular chromatin and occasional conspicuous nucleoli; significant nuclear atypia was not identified. Pertinent negative morphological findings included a lack of Reed-Sternberg cells, vasculitis, or obliterative venulitis. Histochemical stains were negative for fungal organisms and acid-fast bacilli (Grocott-Gomori methenamine silver, Ziehl-Neelsen). By immunohistochemistry, the spindle cells were positive for smooth muscle actin (SMA) (Figure 2B), and did not stain with antibodies targeting cytokeratins, desmin, or ALK. Occasional CD138 plasma cells were present, but there was no absolute increase in IgG4 plasma cells nor was there an increase in the ratio of IgG4 plasma cells to IgG plasma cells. Morphological, histochemical, and immunohistochemical features were considered. In light of the clinical and radiological findings, the features were consistent with a diagnosis of IMT.

In June 2012, the patient presented to the emergency department with acute shortness of breath, fever, and left pleuritic chest pain reportedly similar to his previous admission. New-onset hoarseness was noted. The patient was admitted due to suspicion of a pulmonary embolism. CTPA showed progressive growth of the mass, with new-onset mass effect on the right pulmonary artery also (Figure 1B); there was slight recanalization of the left pulmonary artery. Total body gallium-67 scintigraphy showed a significant local uptake with no evidence of metastatic disease. A PET scan was not done as it would not have impacted clinical management.

Surgical consult deemed the tumor inoperable, and radiotherapy was recommended for the patient. Initially, 20 Gy radiation in 10 fractions was delivered to the left aortopulmonary mass using the 3D conformal technique. Interim lung CT showed no response to therapy, and an additional course of 25 Gy in 15 fractions was delivered to the same area, minimizing dose to the normal tissue, particularly lungs, heart, esophagus, and spinal cord. Thus, a total dose of 45 Gy in 25 fractions was delivered to the mass, which was well tolerated. During radiation, the patient experienced chest tightness, productive cough, and radiation-related esophagitis causing neck pain. His symptoms were controlled via morphine, acetaminophen-oxymorphone combination tablet, and a lidocaine mouthwash.

The patient had a good response to radiotherapy. CTPA taken one month post-treatment displayed a significant reduction in tumor size and further recanalization of the left pulmonary artery lumen (Figure 1C). The patient also noticed an improvement in his shortness of breath and was able to discontinue home oxygen upon completion of his second course of radiotherapy. Pulmonary function test performed in October 2012 showed a FEV1 of 35%, a FVC of 76%, and a FEV1/FVC ratio of 48%. In the years following treatment, he continued to experience exertional shortness of breath, and also noted dysphagia, dysphonia, intermittent dizziness, and bilateral numbness in the hands and feet. Significant dysphagia was treated with dilation. Fourteen months post-radiotherapy, left lower lobe bronchus narrowing was noted via CT, thought to be caused by radiation-related lung fibrosis. Additional surgical consultation post-radiotherapy confirmed that the residual mass remained unresectable.

4.1 years post-treatment, the patient re-presented with dyspnea, cough, and constitutional symptoms; the cause was eventually determined to be a left upper lobe aspergilloma, which was treated with decortication and lobectomy. There was no evidence of myofibroblastic tumor or fibrotic tissues in the pathological specimen. Post-procedure, the patient developed chronic empyema in the left apex that eventually resolved with long-term IV antibiotic therapy and percutaneous insertion of two drains.

Seven years post-treatment, the patient was again admitted for worsening dyspnea and constitutional symptoms, suspected to be due to recurrent aspergillosis. The myofibroblastic tumor was stable on imaging. Ventilation-perfusion imaging indicated severe hypoperfusion (2.6%) of the left lung; therefore the patient was not considered a candidate for further surgical resection. Treatment with IV and inhaled antifungals was initiated, facilitating gradual symptom resolution.

7.3 years post-treatment, the fibrotic mass remains stable (Figure 1D). The patient continues on long-term antifungal therapy, his residual symptoms are well managed with inhalers and prednisone, and his vasculitis is currently in remission. He is followed regularly by Internal Medicine, Respirology, and Rheumatology.

## Discussion

IMT is typically an indolent neoplasm, though local invasion and distant metastasis have been documented in aggressive variants [2]. In addition to the dyspnea and pleuritic chest pain exhibited by this patient, pulmonary IMT can also present with hemoptysis, cough, fever, anemia, and weight loss [1,3]. Approximately one-third of patients with pulmonary IMT are asymptomatic and discovered incidentally [1].

IMT involving the major pulmonary vessels is rare. One case of pulmonary vein stenosis is documented in the literature [5]. Intravascular involvement of the pulmonary veins and arteries has also been reported [6,7]. To our knowledge, this is the first reported case of IMT causing severe (over 80%) pulmonary artery stenosis.

This case afforded a very broad differential diagnosis due to the nonspecific symptoms and complex past medical history. While final pathology favored IMT, the initial differential also included lymphoma, tuberculosis or other infectious cause, sclerosing mediastinitis, IgG4-related pseudotumor, and vasculitis-induced pseudotumor. The patient has been chronically immunosuppressed starting three years prior to diagnosis, which increases risk of fungal and opportunistic infections as well as various rare diagnoses [8]. Cyclophosphamide is known to increase the likelihood of lymphoma and other malignancies in patients with vasculitis, and so consideration was also given to abnormal lymphoma presentation [8].

Appropriate thoracotomy-acquired tissue biopsy and thorough pathological examination were instrumental in narrowing the broad list of possibilities and arriving at a final diagnosis. The specimens exhibited the typical histological findings of IMT and were SMA-positive on immunohistochemistry, a common feature of IMT and other myofibroblastic tumors (Figure 2) [1]. Notably, they were ALK-negative; such a finding does not rule out IMT, as only 50% of cases are positive [1,3]. It subsequently became vital to differentiate between ALK-negative IMT and IgG4-related IPT, as both present with a similar infiltrative pattern and can exhibit SMA positivity [9]. However, IPT demonstrates a significant increase in IgG4-positive plasma cells, both absolute and in relation to IgG-positive plasma cells, and may present with obstructive phlebitis. None of these features were observed in this case, rendering IMT the most likely diagnosis.

It is possible that immunosuppression contributed to the development of IMT in this patient. Cases of IMT have been reported in recipients of lung, renal, and stem cell transplants, occurring both during and after receiving intensive immunosuppressive therapy [10-13]. Harel et al. posited a “two-hit hypothesis,” where immunosuppression acts as an initial “hit,” rendering the individual more vulnerable to subsequent events that promote IMT formation [11]. Proposed second “hits” include infections, such as aspergillosis or hepatitis B virus, and inflammatory conditions, such as inflammatory bowel disease [12-14]. Interestingly, some pediatric cases have shown progression of IMT after corticosteroid administration [15]. Panigada et al. found that fibroblasts derived from these tumors displayed increased proliferation in the presence of dexamethasone, providing further support for a link between immunosuppression and IMT [15].

Numerous treatments have been utilized for unresectable IMTs, including corticosteroids, chemotherapy, radiation therapy, and nonsteroidal anti-inflammatories [16]. Recent trials have suggested that tyrosine kinase inhibitors such as crizotinib, which targets the ALK pathway, may be effective in treating advanced unresectable IMTs in ALK-positive patients [4]. Its role in ALK-negative patients is still unclear. It was decided to proceed with localized radiotherapy in this case, due to the acuteness of the case, a desire to minimize side effects, and the patient’s ALK-negative status. If radiotherapy had not induced a response, tyrosine kinase inhibitors would have been considered.

The literature was referenced to determine appropriate dosage and treatment parameters. For pulmonary IMT unable to be cured with surgical resection and subsequently treated with radiation therapy, reported doses ranged from 40 to 45 Gy in fractions of 0.15-0.21 Gy [17,18]. Radiation therapy used for extrapulmonary IMT often falls within these confines as well [19]. Due to the location of the tumor, a desire to minimize pulmonary side effects, and uncertainty as to the exact pathological diagnosis, it was determined that the treatment would proceed in stages. Initially, 20 Gy in 10 fractions was used to treat to a benign lesion dose in an adaptive treatment plan, which would allow reevaluation if tumor shrinkage was noted. Caution was taken due to concerns regarding pneumonitis due to previous cytotoxic drugs, and attention was paid to the total cardiac dose and other toxicities. However, a lack of response prompted delivery of an additional 25 Gy in 15 fractions to a total dose of 45 Gy as supported by the literature, equivalent to a lymphoma dose [17,18]. This dose was considered sufficient, and in follow-up proved to be effective given the minimal residual fibrotic mass.

## Conclusions

We present a case of unresectable IMT causing left pulmonary artery stenosis in a patient on immunosuppression for p-ANCA vasculitis. Radiotherapy to a total dose of 45 Gy achieved a significant response clinically and on imaging. The differential was broad and included many uncommon entities. Interdisciplinary communication, particularly pathologist input, was essential for diagnosis. We suggest that a registry of these unique cases should be kept for future translational research.
